# Book Review

**Published:** 1894-06

**Authors:** 


*Cancer, Sarcoma, and other Morbid Growths considered in relation to the Sporozoa.* By J. JACKSON CLARKE, M.B. Pp. 97. London: BAILLIÈRE, TINDALL, & COX. 1893.

This book contains an account of the various researches made by many men into the question of the presence of parasitic protozoa in cancer, and in particular a rather egotistical and cock-sure series of assertions as to the author's own observations.

The historical part is well done, containing perhaps the best account in English of the evolution of our knowledge of the subject. The tone of the book is perhaps to be regretted; but a little to be understood on recalling how heavily—and, as recently-published facts seem to show, somewhat unjustly—the Pathological Society came down on the author for some of his statements.

Although the book does not profess to be anything more than a reprint of Journal articles, more use could have been made of it if it had been provided with a table of contents and an index.

*Syphilis: Its Treatment by Intra-muscular Injections of Soluble Mercurial Salts.* By EDWARD COTTERELL, F.R.C.S. Pp. 36. London: JOHN BALE & SONS. 1893.—The author here advocates the treatment of syphilis by the intra-muscular injection of sozoiodol of mercury. He employs a needle of platinoiridium, and uses great care in sterilising it. The injections are made into the gluteal region, and are repeated once a week for about six or seven weeks, and then at less frequent intervals for eighteen months or two years. The objections to this method of treatment are obvious, but there are many compensating advantages.

*Dissections Illustrated.* By C. GORDON BRODIE, F.R.C.S. With plates by PERCY HIGHLEY. In four parts. Part II.—The Lower Limb. Pp. 74. London: WHITTAKER & Co. [1893.]—We have had occasion to notice[1] favourably the first part of this work, and we now welcome the second part as a worthy continuation of the former. The plates of the foot seem to us of special excellence, showing with great accuracy the relative position of the several parts. We can recommend the work to all students of anatomy.

*Guy's Hospital Reports.* Vol. XLIX. Pp. cxxiii., 505. London: J. & A. CHURCHILL. 1893.—The present volume opens with biographical notices of John Hilton and Edward Cock, written by Mr. Jacobson and Mr. Clement Lucas respectively, and illustrated by autotype portraits of the illustrious surgeons of whom they treat. These articles contain much interesting gossip concerning the personal history of Guy's Hospital during the last half-century. Mr. Jacobson had already done much to perpetuate the memory of Hilton by editing two editions of his classical work *Rest and Pain*, and he has now shown still further appreciation of Hilton's genius by writing this account of his life and work. The article does honour alike to author and subject. It is written with all the literary polish with which the writer is so richly endowed, and will be read with the keenest delight by all Hilton's old pupils, as well as by those who only know him as a name and tradition. Mr. Lucas's article brings before us in a graphic form memories of the genial surgeon who has constantly haunted the precincts of Guy's Hospital during the lifetime of the oldest among us, and concerning whom "many a humorous story was told, of which he probably was often as remote an author as Horace or Juvenal; for to a wit of established humour all new witticisms are liable to be attributed by those who dare not father their own offspring, and in the medical world perhaps no one has suffered more than Mr. Cock from this affiliation of bastard stories." Of the purely clinical topics, perhaps the most important is one "On the results of one hundred and thirty cases of Excision of the Knee," by Mr. H. G. Howse, with an analysis of the cases arranged in a tabular form by Dr. G. Newton Pitt. The operations were all performed in the Evelina Hospital and at Guy's during the years 1873 to 1884, and no later cases are given, in order that the results of the operations as shown by the condition of the limbs many years afterwards may be added. Mr. Howse is well known

[1] *Bristol M.-Chir. J.*, vol. xi., 1893, p. 123.

to have excised more knee-joints than any other living surgeon, and wonder has often been expressed that some authentic record of his statistics had not been published; the results of the 130 cases now before us will therefore be read with much interest; and we think Mr. Howse has reason to be gratified with the result of his work. Among the other articles, we would especially call attention to one on "Peritoneal Sanguineous Cysts and their Relation to Cysts of the Pancreas," by Dr. Theodore Fisher, Registrar to the Bristol General Hospital, giving particulars of a great many collected cases of this curious affection.

*Saint Thomas's Hospital Reports.* New Series. Vol. XXI. Pp. xxxviii., 535. London: J. & A. CHURCHILL. 1893. The preface to this volume gives an account of the new buildings at the Medical School attached to the Hospital, on which a sum of £16,000 has been spent. In addition, the physiological laboratory has been rebuilt and refitted, in order to adapt it to requirements for teaching practical physiology to a large class. These additions very materially increase the accommodation at the Medical School, and speak well for the enterprise of the teaching staff. The book contains many valuable papers. There is an interesting memorial notice of the career of one of St. Thomas's most distinguished surgeons, Le Gros Clark. The first paper, a very able account of acute infantile hemiplegia, is from a pen that will alas! write no more. Dr. W. B. Hadden's early death has deprived us of one who promised to attain the highest distinction in the field of neurology. Dr. Cullingworth's valuable paper on "Effusions of the Blood into the Fallopian Tube" is illustrated by some beautiful coloured plates. Dr. Hawkins and Dr. Hector Mackenzie contribute papers on the "Pathology of Perityphlitis" and "Diphtheritic Paralysis" respectively. Drawings of the microscopical appearances are added in the former case, and the paper is a timely contribution to the pathology of this at present much debated affection. Prof. Sherrington gives a useful short note on some points of practical importance connected with the investigation of the knee-jerk. Amongst other papers, there is one by Mr. Makins and Mr. Abbott on the "Treatment of Aneurisms of the Arteries of the Extremities," which is based upon the study of 57 cases. Dr. Percy Smith, in an article on "Sulphonal and Hæmatoporphyrinuria," relates three cases in which this symptom, together with gastro-intestinal disturbance, was directly due to the administration of sulphonal during a considerable period. The more commonly described symptoms of sulphonal poisoning were absent. Dr. Smith thinks that the drug should not be administered "to patients suffering from melancholia, with refusal of food, and evidences of defective intestinal action." Mr. Pitts gives a very interesting account of the "History of Transfusion," of the most speedy method of carrying out this procedure, and of a number of recent cases in which it has been performed. Dr. Sharkey and Mr. Clutton report the successful removal of a pancreatic cyst, and the volume concludes with the usual account of the work done in the medical, surgical, and special departments of the Hospital during the year.

*Sciatic Neuritis.* By Robert Simpson. Pp. 46. Bristol: John Wright & Co. 1893.—This essay sums up what is known of the inflammatory form of sciatica. The normal nutritive changes in the nerves, their degenerative changes, and the reconstructive process are briefly described, and then the nature and action of the various therapeutic agents which favour the restoration of the damaged tissues are considered. Rest, counter-irritation, and massage have given satisfactory results. "By massage, I mean the scientific and skilled manipulations of operators who thoroughly understand what they are doing, and who possess a fine sense of appreciation of the important molecular nutritional and structural changes which they are setting into active operation." Would that we could always secure such massage as this for our patients!

*The Stœchiological Cure of Consumption.* By John Francis Churchill, M.D. Third Edition. Pp. 38. London: David Scott. 1893.—Dr. Churchill appears to adhere to the doctrine that medicine is one of the most backward of all the applied sciences. He endeavours to remove the reproach by making what he conceives to be a great discovery, and after mature reflection he decides that he will not make this public. His decision does not advance the science of medicine, and his methods are beyond the pale of serious criticism.

*A Guide to the Examination of the Urine.* By J. Wickham Legg. Seventh Edition. Edited and Revised by H. Lewis Jones, M.D. Pp. 139. London: H. K. Lewis. 1893.—This edition has been carefully revised, and needs no commendation. Its popularity is the best proof that it is what the student commonly requires; it encourages his abhorrence of too much detail.

*Sciatica.* By A. Symons Eccles, M.B. Pp. 88. London: Macmillan and Co. 1893.—This little book is a record of clinical observations on the causes, nature, and treatment of sixty-eight cases of one or other of the group of sciatic neuralgia, perineuritis or neuritis. Amongst the causes of the malady he does not hesitate to call attention to what is frequently forgotten or ignored, that "of all the products of Western civilisation, the water-closet, such as one finds in the majority of dwelling-houses, may be regarded as one of the most fruitful sources of disease," and he has learned to regard as a necessary addition to the impedimenta of persons who have suffered from sciatica a square piece of felt to cover the seat and slightly overlap the edge, a hole being cut in the centre. He further adds: "Convalescents who are able to leave their beds for the purposes of nature should use a commode draped with a blanket, near whose upper edge a hole has been cut out corresponding in size with the rim of the pan, the blanket being so arranged that it covers the seat of the commode, falls on either side, and forms a mat on the floor in front of the night-stool, so that the patient may step out on the blanket and fold it round the lower extremities, thus avoiding all possibility of exposing the affected limb to sudden or continued cold impressions." The author does not approve of much medication, but prefers treatment by rest, warmth, position and massage, followed by passive and active exercises, for the details of which we must refer the reader to the book itself, on consulting which he will not, we think, be disappointed.

*Étude sur les Abcès chroniques enkystés de l'Amygdale.* Par le Dr. EUG. PEYRISSAC. Pp. 74. Paris: OCTAVE DOIN. 1893.—At the suggestion of Dr. Moure, the author of this pamphlet has collected and reviewed several cases of chronic abscess of the tonsils recently occurring in his laryngological clinic. Though this is a condition which is well worthy of attention, it scarcely merits the separate chapters on the general anatomy and physiology of the tonsil and on the historical aspects of the subject discussed. The author adduces evidence showing that chronic abscess of the tonsil has its origin in former attacks of acute tonsillitis and has no connection with tuberculosis. It is probably the result of infection spreading from a collection of cheesy matter in a tonsillar crypt to the deeper tissues of the tonsil, giving rise to a variety of chronic abscess. The symptoms of the fully-developed condition are, moderate pain, a sense of swelling in the throat, with difficult or even painful deglutition; on examination, one may sometimes observe enormous swelling of the tonsil, which may present the usual characters of a cyst. The clinical notes of ten cases conclude this interesting monograph.

*Higiene de la Educación.* Pp. 62. Barcelona: HENRICH Y C^A, 1893.—This is a "Contestación' or debate on the hygiene of education by Drs. Durán and Bertrán before the Medical Society of Barcelona, and is remarkable for the great length at which the subject is discussed and the suggestions therein contained. Parents and guardians are directed to insist upon perfect hygienic surroundings of schools to which children under their care are sent. It is recommended that gymnasia attached to schools should be in the open air. Much emphasis is laid upon the importance of manual training, a course of which would without doubt be particularly useful to the surgeon. We are told that the task of education is work which is specially patriotic, because its mission is to form men and to form citizens, and as the next generation is the hope of a country every care should be taken that the hope may be realised.

*Burdett's Hospital and Charities Annual*, 1894. Edited by HENRY C. BURDETT. London: THE SCIENTIFIC PRESS (LIMITED.)—This book has proved itself a necessity for all who in this busy age require without much laborious searching a record of general and statistical information of British, American and Colonial hospitals and asylums, medical schools and colleges, religious and benevolent institutions, dispensaries, nursing and convalescent institutions. This information would have been still more valuable if Mr. Burdett had received more help from the officials of institutions, many of whom, we are sorry to see, have not afforded him the information which is necessary to make a book of this kind perfect. Mr. Burdett is always on the war-path on the question of the cost of hospital management, and he devotes a large space of this volume to its consideration. Mr. Burdett is evidently one who pays some attention to the reviews of his work, and we are therefore surprised to find that he has repeated, although notice was directed to it last year, the error of the relation of the Medical School to the Bristol Royal Infirmary and the Bristol General Hospital.

*The Medical Annual*. 1894. Bristol: JOHN WRIGHT & Co.—This volume, which is now in its twelfth year, is full of interesting reading, and does great credit to the various editors and contributors who are responsible for it. It is practically indispensable to the busy medical practitioner who has not the leisure necessary for reading the original articles of which this is a *resumé*, as it gives him at a glance most of the suggestions for treatment that have been published during the year. Both in the quality of the matter and in the way it is put before us by the publishers it is a worthy successor to the volumes that have preceded it. It is not surprising that we have noticed some mistakes in spelling the numerous names of authors quoted; *e.g.*, described as Brysan Delevan and Goodheart, the persons referred to would scarcely know themselves. We are glad to see that Bristol still takes part in its authorship in the persons of Drs. Shingleton Smith and Watson Williams. It is a pity that the advertisements are placed as they are. In future issues none of these should be allowed to come between the title-page and the last page of the medical portion of the volume.

*Proceedings of the West London Medico-Chirurgical Society*. Vol. V. Pp. 122. London: BAILLIERE, TINDALL AND COX. 1893.—In addition to many papers of considerable interest, this volume contains the Cavendish Lectures on "Elimination and its Uses in Preventing and Curing Disease," by Dr. Lauder Brunton, and on "Some Points in the Etiology of Typhoid Fever," by Sir C. Cameron. Not the least interesting of the papers is that by Dr. Towers Smith on the treatment of Obesity. Our West London brethren seem to be rather behind the times. In this, their latest, volume, the most recent communication is dated July 8th, 1892, and the earliest is October 3rd, 1890. Surely a society which numbers 330 and prides itself on the value and character of its work might issue an annual volume. A little more careful editing is also needed. On the cover the work is called "Transactions," and on the title-page "Proceedings." There is no table of contents, and Dr. Lauder Brunton's lecture is most curiously described in its title.

*The Johns Hopkins Hospital Reports.* Vol. III. Nos. 7, 8, 9. Report in Gynæcology, II. Baltimore: The Johns Hopkins Press. 1894.—This volume contains many interesting and valuable articles on gynæcological work, including cases where cœliotomy is required. The illustrations are numerous and well executed, and the statistics are carefully compiled. To show the extent and usefulness of the work here recorded, we have only to mention the analysis of 240 cases showing the importance of anæsthesia in the diagnosis of doubtful conditions of the pelvic organs, and a table of 512 abdominal operations since the last volume was issued. The report reflects much credit on its contributors and editors.

*Index-Catalogue of the Library of the Surgeon-General's Office, United States Army.* Vol. XIV.: Sutures—Universally. Washington: Government Printing Office. 1893.—We have to thank Dr. John S. Billings for another volume of this great work, without which no medical library can be looked upon as complete. This volume contains 10,124 author titles, representing 6,426 volumes and 8,850 pamphlets. The whole series of 14 volumes contains the gigantic number of 151,649 book titles and 462,165 journal articles. We congratulate the painstaking authors on the fact that they are so near the end of the alphabet, and ourselves that the work is sufficiently complete to be looked upon as a trustworthy friend who rarely fails to respond to any call.

*When may Syphilitics Marry?* By Dr. Schuster. Edited and Translated by C. Renner. London: F. J. Rebman. 1893. —It is most difficult to say when syphilis is really cured, when the patients will never again show any symptoms or transmit the disease. The work before us is an earnest effort to form sound conclusions before answering the question put. Many of the recognised authorities are quoted, and the author weighs carefully their arguments, and states his own views in a clear and logical way. This little book is worthy of careful perusal. It has in Germany reached six editions in a comparatively short time.

*The Treatment of Constitutional Syphilis.* By Oswald Ziemssen, M.D. Pp. 70. London: H. K. Lewis. 1893.—This booklet contains some useful hints, amongst them that the individual affected by syphilis should remain under the care of one medical man throughout the disease, even if it be at times advisable to consult a specialist for some peculiar phase of the affection. The author considers that, like other infectious diseases, syphilis is caused by a microbe, and that the aim of treatment should be to kill the microbe or modify the soil in which it flourishes. "General treatment—curative, not preventive—should be commenced as soon as possible after infection." Mercury, of course, is the curative agent, the maximum dose being different for different patients. It is essential that the patient should have plain nourishing food, avoid all exertion, and live in fresh air. "The acknowledged mildness of the climate of Wiesbaden [where the author lives] makes it particularly suited for the purpose;" and as Wiesbaden also possesses baths, the author is in favour of thermal treatment associated with inunction of mercury. He considers that mercury cures the disease itself, while the iodide of potassium only causes the absorption of the products of the disease. There seems little that is original in the volume, which can scarcely be looked upon as a scientific treatise. It has neither table of contents nor index.

*Teratologia.* By J. W. BALLANTYNE, M.D. No. 1. April, 1894. London: WILLIAMS & NORGATE.—Those who have read Dr. Ballantyne's able work, *Diseases and Deformities of the Fœtus*, will welcome this quarterly journal, in the first number of which he has a valuable and well-illustrated paper on "The Fœtus Amorphous." A very interesting feature of the work is the portion devoted to the "Abstracts from Current Literature," which gives references to all recent writings on Teratology and in many cases refers to important points in these communications. The most interesting monstrosity described in the journal is that of the Devil himself. The mother during her pregnancy said, in a rage, "that she would rather have the Devil in the house than something offered her for sale;" and so the Devil came, and a very typical Devil he turned out to be, probably one whose anatomy would be interesting. We hope to have a description of it in a future number, but unfortunately he is still living. We need hardly say that the incident is described under the heading "Survival of Superstitious Beliefs with regard to Teratological Phenomena," and is only an amusing appendix to a very valuable journal.

*The Students' Handbook of Gynecology.* Edinburgh: E. & S. LIVINGSTONE. 1893.—The attempt to provide in 178 pages of small size and large type a "Handbook" of Gynecology must be foredoomed to failure. In this volume the result and the attempt are both bad. It is to be hoped that it will never find its way into the hands of any student or medical man seeking for light on this important branch of our professional work. The reader must not be altogether disappointed with the size of this volume; for in addition to the 178 pages of gynecology, the kindness of the publishers has supplied him with 36 pages of advertisements. We are not surprised to find that the author, contemplating his finished work, decided not to put his name to it; but he might have given it an index.

*The Mineral Waters of Harrogate.* By JOHN LIDDELL, M.D. Pp. iv., 62. Edinburgh: YOUNG J. PENTLAND. 1893.—"The celebrity which Harrogate has acquired by its medicinal springs tends to overshadow the capacities of the place as a health-resort." It has already acquired so good a reputation that it needs no puffing. It is nevertheless necessary that a knowledge of the chief characteristics of the climate and the waters of the eighty springs should be imparted to medical men who advise their patients to seek treatment in this region, and a perusal of this book will show that Harrogate occupies not only the highest position among the spas of England, but that it stands unequalled in Europe for variety of waters and their therapeutic range.

*Alcohol and Public Health.* By J. JAMES RIDGE, M.D. Second Edition. London: H. K. LEWIS. 1893.—A second edition of this book calls for little comment. Dr. Ridge's views on this subject, and the behaviour of his geranium cuttings, are now well known, and he maintains "that it is obligatory on the part of the defenders of alcohol-drinking to prove that it is harmless; and that till this is done, we should advise total abstinence as a certain means of preventing all the evils, small or great, which result from its use."

*Introduction to the Catalogue of the Collection of Calculi of the Bladder.* By Sir HENRY THOMPSON. Pp. 39. London: J. & A. CHURCHILL. 1893.—In the summer of 1892, Sir Henry Thompson presented his collection of calculi—upwards of one thousand in number—to the Hunterian Museum of the Royal College of Surgeons. A catalogue was presented at the same time, and the present work forms an introduction to this catalogue, giving some interesting particulars of the various operations performed by the author, together with statistics of mortality and other tables and details. Sir Henry Thompson has done more than any other living British surgeon to improve and perfect the treatment of stone in the bladder, and this record of his life's work will remain as evidence of his genius and industry.

*Ancient Egyptian Medicine. Herophilus and Erasistratus.* By JAMES FINLAYSON, M.D. Glasgow: ALEX. MACDOUGALL. 1893. —We were very glad to have in separate form these admirable papers, which we previously had the pleasure of seeing when they were published respectively in the *British Medical Journal* and the *Glasgow Medical Journal.* They were delivered as bibliographical demonstrations in the Library of the Faculty of Physicians and Surgeons of Glasgow, and form fitting continuations of Dr. Finlayson's demonstrations on Hippocrates, Galen, and Celsus. The papers are of great interest and are marked by considerable erudition. Fortunate is the institution that has Dr. Finlayson as its librarian. We hope to receive many similar contributions from his scholarly pen.

*Science Progress.* A Monthly Review of Current Scientific Investigation. Conducted by HENRY C. BURDETT. Edited by J. BRETLAND FARMER. Vol. I. No. 1. March, 1894. London: THE SCIENTIFIC PRESS LIMITED.—This periodical is due to the energy and liberality of Mr. Burdett. We say "liberality," as we should think it doubtful whether it will pay. This number contains a series of articles on various branches of science by recognised authorities, giving an account of recent progress along a particular line of investigation. The articles vary a good deal in interest—*e.g.* Dr. Halliburton's paper on "Chemical Physiology" is rather dry and a little like a collection of notes from a *Centralblatt*, whilst that of Mr. Howes on "Vertebrate Morphology" is written in an enthusiastic tone. The paper on "The New Theory of Solutions" is perhaps the most interesting; in it Mr. Rodger shows that recent researches point to the view that in dilute solutions, dissolved substances behave as if they were gaseous and diffused through the space occupied by the solvent. The difficulty of the periodical must be that the articles may become so specialised that most men will not be able to read and enjoy more than two or three in each number; but at present it is well worth buying.

*Transactions of the Clinical Society of London.* Vol. XXVI. Pp. xlvi., 265. LONGMANS, GREEN AND CO. 1893.—This volume has most interesting matter on a great range of subjects. Two papers on the subject of intra-thoracic suppuration from perforation of gastric ulcer, and one on double empyema are of especial interest.

*Essentials of Minor Surgery, Bandaging, and Venereal Diseases.* By EDWARD MARTIN, M.D. Second Edition. Pp. 166. Philadelphia: W. B. SAUNDERS. 1893.—We are not very much in favour of the question and answer method of imparting instruction; but we have nothing but praise for the subject-matter of this book, which is evidently appreciated, as it has quickly reached a second edition. There are 78 illustrations, most of which are very clear and good, while the letterpress is well written and to the point. The principal matters usually dealt with in works on minor surgery are well treated; but we fail to find any description of the numerous minor operations usually performed by house-surgeons or dressers, such as removal of foreign bodies from the eye and ear, plugging the nares, the use of the stomach-pump, &c. In our opinion, it would have been more useful, in such a work as this, to have supplied these omissions instead of filling up the book with a description of venereal diseases, which scarcely seems in the right place here. We can only suppose that the explanation of the combination is to be found on the title-page, where we notice that the author is a clinical professor of genito-urinary diseases, and at the same time lecturer on minor surgery in the University of

Pennsylvania. The type, paper, binding, &c., are up to the usual high standard of American excellence.

*The Hygienic Prevention of Consumption.* By J. EDWARD SQUIRE, M.D. Pp. xii., 194. London: CHARLES GRIFFIN AND COMPANY. 1893.—Dr. Squire has very wisely recognised the fact that the prevention of consumption is in part a question of public, and in part also a question of individual, hygiene. The general principles of preventive hygiene from infancy and childhood to adult life are dealt with in chapters which contain much careful and well-considered advice: school life, exercise, clothing and diet, all receive adequate notice, and the chapter on prevention of consumption in the family when one member of the household is consumptive, strikes us as of especial value. We think that the author's wish that the book may be acceptable to his professional brethren, as well as to large numbers of others interested, is likely to be realised.

*Aids to Otology.* (Second edition of *Epitome of Ear Diseases.*) By W. R. H. STEWART. Pp. 110. London: BAILLIERE, TINDALL, AND COX. [1893.]—We are warned in the preface that "this book is in no way intended to take the place of the larger works on ear-disease," but we scarcely think such a warning necessary; for it must be obvious to the most cursory reader that such a book as this could hardly convey any real information, much less take the place of a scientific work. We presume the intention of the author has been to supply a short and easy book of reference which should be useful to anyone who had not already acquired a knowledge of ear diseases, but we fear that in his anxiety to be brief he has spoilt any usefulness which the book might have possessed. For example, under the head of Othæmatoma we find, "*treatment*, evaporating lotions; vesication; puncture of tumour and aspiration; evacuating contents and applying pressure; ice, iodine; lead lotion; methodical massage." How is the unfortunate surgeon, seeking here to find the best treatment to pursue, to get any help from such a jumble as this? There is a total absence of literary style, and such sentences as the following abound: (p. 57) "The hearing power is the last symptom to get well;" while among the numerous errors, we notice (p. 10, line 10) auricular temporal for auriculo-temporal, (p. 13, line 22) cruræ for crura, and (p. 52, line 8) inflammation of middle ear for inflammation of drum membrane.

*Myxœdema, and the Effects of Climate on the Disease.* By A. MARIUS WILSON, M.D. Pp. 36. London: THE SCIENTIFIC PRESS, LIMITED. 1894.—We entirely fail to see the *raison d'être* of booklets of this sort. This one consists of 36 small pages in the largest of type on the thickest of paper. It adds nothing fresh to our knowledge of myxœdema, and does not even give a good account of what is already known about it: a better one will be found in any of the ordinary text-books.

*A Practical Treatise on Diphtheria and its Successful Treatment.* By BROWNLOW R. MARTIN, M.B. Pp. 32.—London: BAILLIERE, TINDALL AND COX. 1894.—A treatise on diphtheria in 32 pages of large print, the book also containing 42 pages of advertisements in small type! The author tries to disarm criticism by the humility of his preface; we will therefore content ourselves with saying that he believes that sulphite of magnesium locally applied to the fauces will abort or cut short diphtheria, and that, in our opinion, he brings forward no sufficient proof of his thesis.

*Diseases of the Skin.* By MALCOLM MORRIS. Pp. xii., 556. CASSELL AND COMPANY, LIMITED. 1894.—We have much pleasure in reviewing this work, because we believe the author is actuated by high motives, and is desirous of extending the knowledge of these intricate diseases by careful observation and sound investigation. Great advances have been made of late years in dermatology, and the work is well up to date. The coloured plates and drawings are very well done, and are a valuable addition. The former are well chosen, and they present very good contrasts of diseases which may easily be confounded. In Plate IV. the ear eczemas and lupus erythematosus of the same organ are beautifully compared; but we have a feeling that the lupus case will become one of vulgaris, even if there has not been already ulceration and scarring. The chapters on "Bacteriology" and "Classification" are well and clearly written, as is also the one on "Principles of Diagnosis;" but in making the distinction between varicella and variola (a discrimination very important in these days, when there are so many qualified men who, thanks to vaccination, have rarely or never seen a case of small-pox), we notice a very important omission; viz., that in chicken-pox, however severe the case may be—and therefore the more liable to be confounded with small-pox,—careful examination will always reveal the rash in, at least, the three stages of clear vesicle, a pustule, and a drying-up scab simultaneously, and often in close contiguity. This never happens in small-pox. The correct diagnosis is most important, because a mild attack of variola may be the source of infection in a very severe case, whilst chicken-pox can only produce chicken-pox. The description and treatment of eczema occupies a very important portion of the work. Mr. Morris has advocated for some time the use of tartar emetic in the treatment of some of these troublesome cases, and we can endorse the plan; but we are told there is very little new under the sun, and we find in an edition of Pareira for 1849, the bisulphate and peroxide of antimony spoken of rather highly as skin remedies. Now tartar emetic depends chiefly for its activity upon the large amount of the peroxide of antimony, which it contains. We are glad to find "acne" placed amongst the "Local Inoculable Diseases," as we have been impressed for some time with the view that acne depends upon a "germ,"

probably a micro-organism, and the most successful treatment embodies this idea. In speaking of Psoriasis (p. 271), the author states there are *no crusts;* Crocker defines it as "scaly crusts on a red base," and we think this definition as generally correct—short and pithy. In the chapter on Tinea, Sabouraud's views are clearly stated; but we have had several cases of T. tonsurans in members of the same family, and having, apparently, a common origin, and yet the results of similar treatment have been very varying; but no scalps are physiologically the same, and the difference may be accounted for by this rather than another species of fungus. There are many other points we should like to notice, but space forbids. We can commend the work most thoroughly to practitioners and students. It is well got up, and is exceedingly moderate in price.

*A Guide to the Public Medical Services.* By Alexander Faulkner, Surgeon-Major Indian Medical Service. London: H. K. Lewis. 1893.—This pamphlet is what it professes to be —a work of reference. It will be found a useful guide for any aspirant who desires to join any of the following Public Medical Services: The Royal Navy, Medical Department of the Army, West Coast of Africa Medical Service, Colonial Medical Service, the Home Civil Medical Service, such as The General Post Office, Local Government Board, Factory Department, Dispensary, Workhouse and other Government Medical Appointments in Ireland. The information contained in the work may be thoroughly depended on to give accurate information, at all events up to November, 1893, as it contains little but exact copies of the official regulations published from the headquarters of the various services above mentioned. Everything that a candidate for one of these appointments requires to know will be found in the pages of Surgeon-Major Faulkner's little work.

*A Practical Handbook of Midwifery.* By Francis W. Nicol Haultain, M.D. Pp. 248. London: The Scientific Press Limited. 1894.—This book fulfils the object of its author in that it places before the student and practitioner the chief points in the study of obstetrics; but it certainly would be too concentrated for anyone who has not either heard the author's lectures or read some more detailed account of the subject. The plan of the work is good for reference, and the book itself contains much valuable information. The chapter on the "Signs of Pregnancy" is very well written, and that on "The Pathology of Pregnancy" is very comprehensive, but we cannot accept all the author's statements in reference to the effects of pregnancy on some morbid conditions. The excellent way in which Dr. Haultain deals with the diseases due to pregnancy gives a distinct stamp to the book. He states that in retroflexion of the gravid uterus the bladder trouble comes on after the fourth month; our experience is that in such cases it is most frequently at the end of the third, that there is difficulty in micturition, and often retention of urine. Dr. Haultain has many practical hints on the management of labour, and we agree with him in his preference for the axis traction forceps of Milne Murray. The other portions of the book are full of information, and would well repay anyone reading them while waiting at a midwifery case. The book is especially one to refresh one's memory from, rather than a student's book to teach him the subject.

---

We have received from Messrs. J.-B. Baillière et Fils a copy, dated May, 1894, of their "Bibliographie Méthodique des Maladies de l'Enfance." Upon request, addressed to them at 19 Rue Hautefeuille, Paris, the publishers will send this very useful catalogue to any one desirous of having it.

Commencing with the July issue, the *Archives of Pediatrics* (Bailey & Fairchild, New York) will be edited by Dr. Dillon Brown, Adjunct Professor of Pediatrics at the New York Clinic.

---

